# Supplementary material for: Disentangling structure-function relationships between the human hippocampus and the whole brain using track-weighted dynamic functional connectivity
Source: bioRxiv. 2025 Oct 20:2025.10.19.683338. Preprint. [Version 1] doi: 10.1101/2025.10.19.683338 (PMC12633360; doi:10.1101/2025.10.19.683338)
Supplement: 1 [file NIHPP2025.10.19.683338V1-supplement-1.pdf]

bioRxiv preprint doi: https://doi.org/10.1101/2025.10.19.683338; this version posted October 20, 2025. The copyright holder for this preprint (which was not certified by peer review) is the author/funder, who has granted bioRxiv a license to display the preprint in perpetuity. It is made available under aCC-BY 4.0 International license.

Tables.

Supplementary Table 1. Results of the Study 2 rsfMRI comparisons. Results are reported at P<0.05 FDR corrected.

			MNI Peak Co-ordinate			
Contrast	Location of Peak Voxel	Hemi-sphere	x	y	z	Number of Voxels
Cluster A > Cluster B	Cerebellum	L	-30	-70	-56	20641
	Dorsal anterior cingulate gyrus	L	-6	20	40	5101
	Lateral fronto-orbital gyrus	L	-22	50	-14	505
	Inferior occipital gyrus	L	-50	-62	-4	342
	Posterior middle temporal gyrus	R	58	-50	-8	318
	Superior frontal gyrus (posterior segment)	L	-14	2	68	293
	Middle frontal gyrus (dorsal prefrontal cortex)	R	24	52	24	173
	Superior frontal gyrus (posterior segment)	R	6	30	44	102
	Fusiform gyrus	L	-32	-34	-10	26
	Dorsal anterior cingulate gyrus	L	-2	22	26	24
	Postcentral gyrus	L	-20	-32	62	20
Cluster B > Cluster A	Precentral gyrus	L	-6	-24	62	2514
	Fusiform gyrus	R	32	-32	-12	1976
	Amygdala	R	-20	-6	-26	1940
	Postcentral gyrus	R	6	-26	58	1725
	Subgenual anterior cingulate gyrus	R	4	12	-10	652
	Cerebellum	R	34	-76	-34	556
	Cerebellum	L	-14	-88	-38	387
	Inferior frontal gyrus pars opercularis	L	-52	20	18	344
	Cerebellum	R	6	-52	-42	294
	Inferior frontal gyrus pars orbitralis	R	48	36	-12	255
	Precuneus	R	6	-64	44	139
	Precuneus	L	-10	-54	34	123
	Posterior cingulate gyrus	R	16	-54	18	97
	Middle occipital gyrus	L	-36	-78	38	85
	Posterior cingulate gyrus	L	-6	-54	10	84
	Angular gyrus	R	52	-50	24	61
	Supramarginal gyrus	R	48	-18	18	61
	Precentral gyrus	R	40	-6	16	40
	Fusiform gyrus	L	-42	-48	-18	38

bioRxiv preprint doi: https://doi.org/10.1101/2025.10.19.683338; this version posted October 20, 2025. The copyright holder for this preprint (which was not certified by peer review) is the author/funder, who has granted bioRxiv a license to display the preprint in perpetuity. It is made available under aCC-BY 4.0 International license.

	Supramarginal gyrus	L	-42	-26	20	37
	Middle occipital gyrus	R	48	-70	8	29
	Posterior middle temporal gyrus	L	-50	-54	20	25
	Middle occipital gyrus	L	-46	-76	12	25
Cluster A > Cluster C	Superior frontal gyrus (posterior segment)	R	8	10	48	5594
	Precentral gyrus	L	-28	-6	50	140
	Superior frontal gyrus (posterior segment)	L	-18	14	62	130
	Anterior cingulate gyrus	R	6	40	6	105
	Inferior temporal gyrus	L	-52	-10	-32	102
	Inferior temporal gyrus	R	48	-2	-34	96
	Entorhinal/fusiform cortex	R	24	-4	-34	53
	Entorhinal/fusiform cortex	L	-24	-4	-36	41
	Postcentral gyrus	L	-20	-32	62	29
	Superior frontal gyrus (prefrontal cortex)	L	-10	52	2	25
	Rostral anterior cingulate gyrus	L	-4	42	4	22
	Posterior middle temporal gyrus	L	-52	-28	-6	20
Cluster C > Cluster A	Precentral gyrus	R	36	-22	54	1518
	Postcentral gyrus	L	-62	-10	30	1063
	Superior frontal gyrus (prefrontal cortex)	R	6	50	22	558
	Postcentral gyrus	L	-10	-28	72	388
	Superior frontal gyrus (prefrontal cortex)	L	-6	60	18	364
	Cerebellum	R	6	-50	-46	348
	Gyrus rectus	L	-4	22	-12	252
	Cerebellum	R	40	-72	-36	167
	Cerebellum	R	14	-86	-38	166
	Cerebellum	L	-12	-86	-40	165
	Inferior frontal gyrus pars triangularis	L	-46	24	20	132
	Precuneus	R	6	-64	46	122
	Cerebellum	L	-38	-72	-36	122
	Precuneus	L	-8	-54	40	103
	Inferior frontal gyrus pars orbitralis	L	-40	38	-14	100
	Precuneus	L	-16	-62	22	56
	Angular gyrus	R	52	-54	18	54
	Posterior middle temporal gyrus	L	-60	-44	-8	53
	Middle occipital gyrus	R	38	-76	36	52
	Supramarginal gyrus	R	42	-24	20	48
	Middle frontal gyrus (posterior segment)	R	48	28	18	44
	Inferior frontal gyrus pars orbitralis	R	38	40	-12	41
	Superior frontal gyrus (posterior segment)	L	-4	4	66	41
	Inferior frontal gyrus pars orbitralis	R	42	30	-14	30
	Middle occipital gyrus	L	-46	-76	12	29
	Supramarginal gyrus	L	-44	-24	20	28
	Precentral gyrus	R	40	-6	16	28
	Subgenual anterior cingulate gyrus	R	2	22	-8	28
	Posterior middle temporal gyrus	R	62	-40	-8	27
	Superior occipital gyrus	R	22	-60	22	22
Cluster B > Cluster C	Cerebellum	L	-42	-56	-44	6945

bioRxiv preprint doi: https://doi.org/10.1101/2025.10.19.683338; this version posted October 20, 2025. The copyright holder for this preprint (which was not certified by peer review) is the author/funder, who has granted bioRxiv a license to display the preprint in perpetuity. It is made available under aCC-BY 4.0 International license.

	Posterior cingulate gyrus	R	4	-26	40	6030
	Anterior insular	L	-38	12	-10	1687
	Lateral fronto-orbital gyrus	L	-26	44	-12	784
	Supramarginal gyrus	L	-56	-42	40	454
	Angular gyrus	R	46	-56	46	433
	Middle temporal gyrus	R	52	-24	-8	412
	Posterior middle temporal gyrus	L	-52	-32	-4	336
	Anterior middle temporal gyrus	L	-52	-6	-30	322
	Inferior temporal gyrus	R	50	4	-34	280
	Middle occipital gyrus	R	30	-92	2	136
	Superior occipital gyrus	R	16	-64	30	117
	Putamen	L	-26	-2	8	106
	Medulla	R	4	-40	-44	83
	Middle occipital gyrus	L	-36	-88	-6	73
	Superior parietal gyrus	L	-24	-48	64	63
	Anterior fusiform gyrus	R	24	-8	-36	47
	Cuneus	L	-6	-82	34	36
	Pallidum	R	16	-4	-4	35
	Superior parietal gyrus	R	28	-44	64	33
	Brain stem, pontine crossing tract	R	6	-24	-28	32
	Cuneus	R	12	-80	12	29
	Cerebellum	R	28	-34	-37	26
	Cuneus	L	-12	-74	42	24
Cluster C > Cluster B	Posterior cingulate gyrus	L	-6	-56	12	271
	Gyrus rectus (vmPFC)	L	-8	46	-10	265
	Posterior cingulate gyrus	R	6	-58	16	264
	Posterior fusiform gyrus	L	-24	-32	-20	246
	Gyrus rectus (vmPFC)	R	4	38	-18	228
	Posterior fusiform gyrus	R	28	-30	-18	227
	Superior frontal gyrus (posterior segment)	L	-22	28	40	221
	Superior frontal gyrus (posterior segment)	R	26	20	46	162
	Middle occipital gyrus	L	-38	-80	32	158
	Angular gyrus	R	44	-70	36	133
	Parahippocampal gyrus / amygdala	L	-22	-4	-28	128
	Amygdala	R	18	-8	-17	124
	Cerebellum	L	-10	-86	-38	113
	Middle temporal gyrus	R	54	-6	-18	109
	Precentral gyrus	R	6	-26	62	91
	Middle frontal gyrus (posterior segment)	L	-44	28	18	79
	Cerebellum	R	7	-51	-47	79
	Inferior frontal gyrus pars orbitralis	L	-38	34	-12	71
	Cerebellum	L	-8	-46	-42	63
	Middle temporal gyrus	L	-60	-8	-18	63
	Lateral fronto-orbital gyrus	R	34	34	-12	60
	Cerebellum	R	14	-86	-36	51
	Precentral gyrus	L	-10	-20	72	37
	Posterior middle temporal gyrus	L	-58	-46	-10	35
	Posterior fusiform gyrus	R	42	-28	-22	33
	Middle frontal gyrus (posterior segment)	R	44	32	18	30

bioRxiv preprint doi: https://doi.org/10.1101/2025.10.19.683338; this version posted October 20, 2025. The copyright holder for this preprint (which was not certified by peer review) is the author/funder, who has granted bioRxiv a license to display the preprint in perpetuity. It is made available under aCC-BY 4.0 International license.

	Postcentral gyrus	L	-4	-34	60	26
	Pole of middle temporal gyrus	R	42	16	-34	24
	Postcentral gyrus	R	42	-14	56	22

bioRxiv preprint doi: https://doi.org/10.1101/2025.10.19.683338; this version posted October 20, 2025. The copyright holder for this preprint (which was not certified by peer review) is the author/funder, who has granted bioRxiv a license to display the preprint in perpetuity. It is made available under aCC-BY 4.0 International license.

Appendices

Supplementary Video 1. ***Representative TW-dFC map from a single participant.*** *This video provides a compelling visualisation of time-resolved hippocampal connectivity, capturing dynamic fluctuations in how the hippocampus functionally engages with the broader brain over time. Functional coupling is projected onto the white matter pathways that mediate these interactions, illustrating how specific anatomical tracts mediate shifting patterns of communication between the hippocampus and distributed cortical and subcortical regions. Notably, the hippocampus exhibits time-varying interactions with occipital, parietal, temporal, frontal, and thalamic areas throughout the resting-state scan. This dynamic mapping underscores the temporally evolving nature of hippocampal network integration, shaped and constrained by its structural connectivity.*

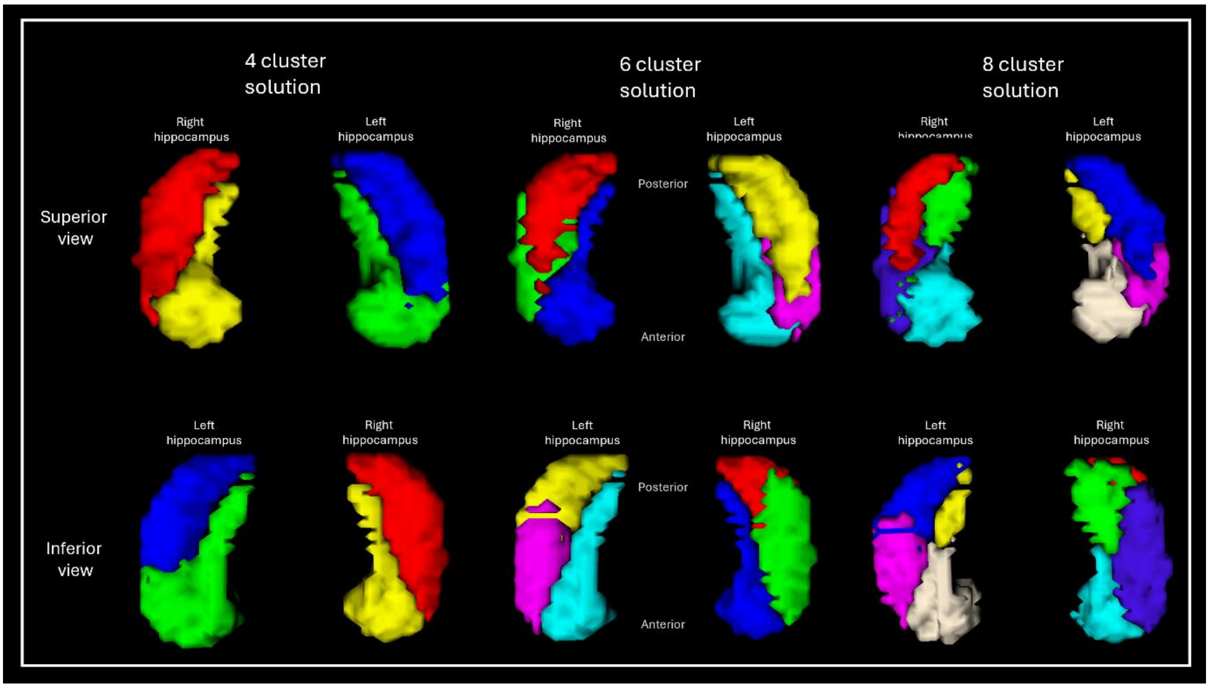

Supplementary Figure 1. 3D renderings of hippocampal clusters identified via k-means clustering of independent component analysis (ICA) on TW-dFC data, shown for 4-, 6-, and 8-cluster solutions. *Clusters are distributed along both the anterior-posterior (anterior towards the bottom of the figure, posterior towards the top of the figure) and medial-lateral axes of the hippocampus. 4-cluster solution (left); Rather than a simple anterior-posterior split, this solution revealed two spatially distinct divisions within each hippocampus: an anteromedial division encompassing the uncal and medial portions (yellow and green clusters), and a posterolateral division encompassing the hippocampal tail and lateral portions (red and blue clusters). 6-cluster solution (middle); This parcellation is described in detail in the main text and Figure 2. 8-cluster solution (right); Similar in organisation to the 6-cluster solution but displayed a further subdivision of the medial cluster into anterior (cyan and beige) and posterior (green and yellow) components. Across all solutions, note the striking bilateral symmetry in cluster organisation across the left and right hippocampus, despite no spatial constraints having been imposed during clustering.*

bioRxiv preprint doi: https://doi.org/10.1101/2025.10.19.683338; this version posted October 20, 2025. The copyright holder for this preprint (which was not certified by peer review) is the author/funder, who has granted bioRxiv a license to display the preprint in perpetuity. It is made available under aCC-BY 4.0 International license.

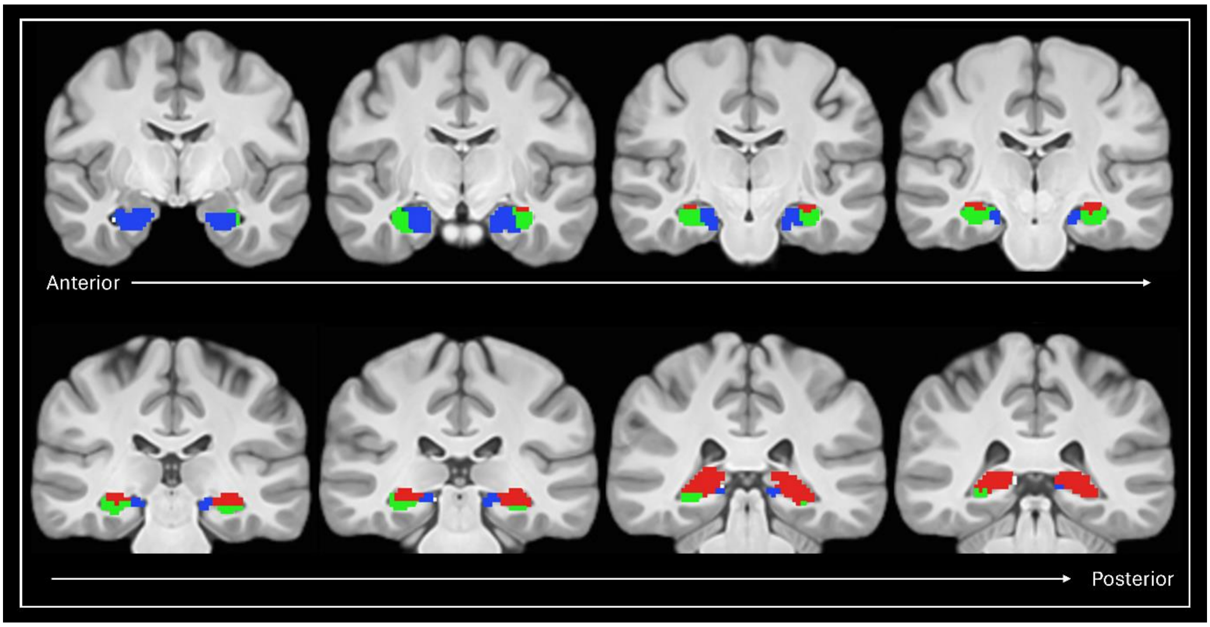

Supplementary Figure 2. Anterior-posterior distribution of TW-dFC-derived clusters. *Clusters identified using the 6-cluster TW-dFC solution are overlaid on coronal slices of a T1-weighted image progressing from the anterior (top left) to posterior (bottom right) extent of the hippocampus. Within the hippocampal body, individual clusters show broad spatial correspondence with cytoarchitecturally defined subfields (described in text and visually presented in Figure 2B). In contrast, broader functional clusters were observed in the hippocampal head and tail, encompassing multiple subfields and suggesting coarser functional organisation in these regions.*

bioRxiv preprint doi: https://doi.org/10.1101/2025.10.19.683338; this version posted October 20, 2025. The copyright holder for this preprint (which was not certified by peer review) is the author/funder, who has granted bioRxiv a license to display the preprint in perpetuity. It is made available under aCC-BY 4.0 International license.

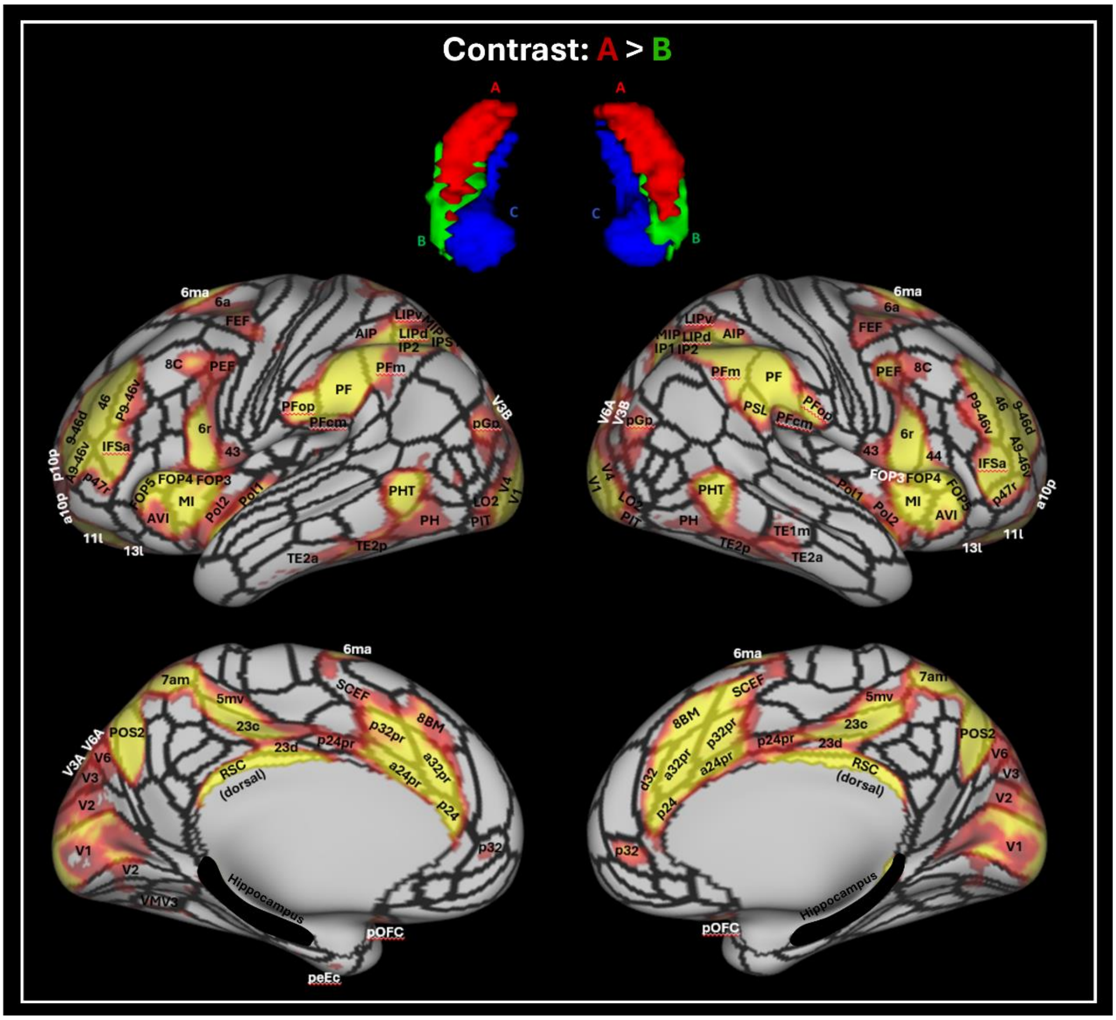

Supplementary Figure 3. *Results of the seed-based rsfMRI analysis based on the TW-dFC hippocampal clusters.* *Results for the contrast of cluster A (hippocampal tail cluster) > cluster B (anterolateral cluster). Results are overlaid on the HCPMMP (hippocampus is highlighted in black) and statistically significant areas are labelled. T-test results are thresholded at p<0.05 FDR corrected.*

bioRxiv preprint doi: https://doi.org/10.1101/2025.10.19.683338; this version posted October 20, 2025. The copyright holder for this preprint (which was not certified by peer review) is the author/funder, who has granted bioRxiv a license to display the preprint in perpetuity. It is made available under aCC-BY 4.0 International license.

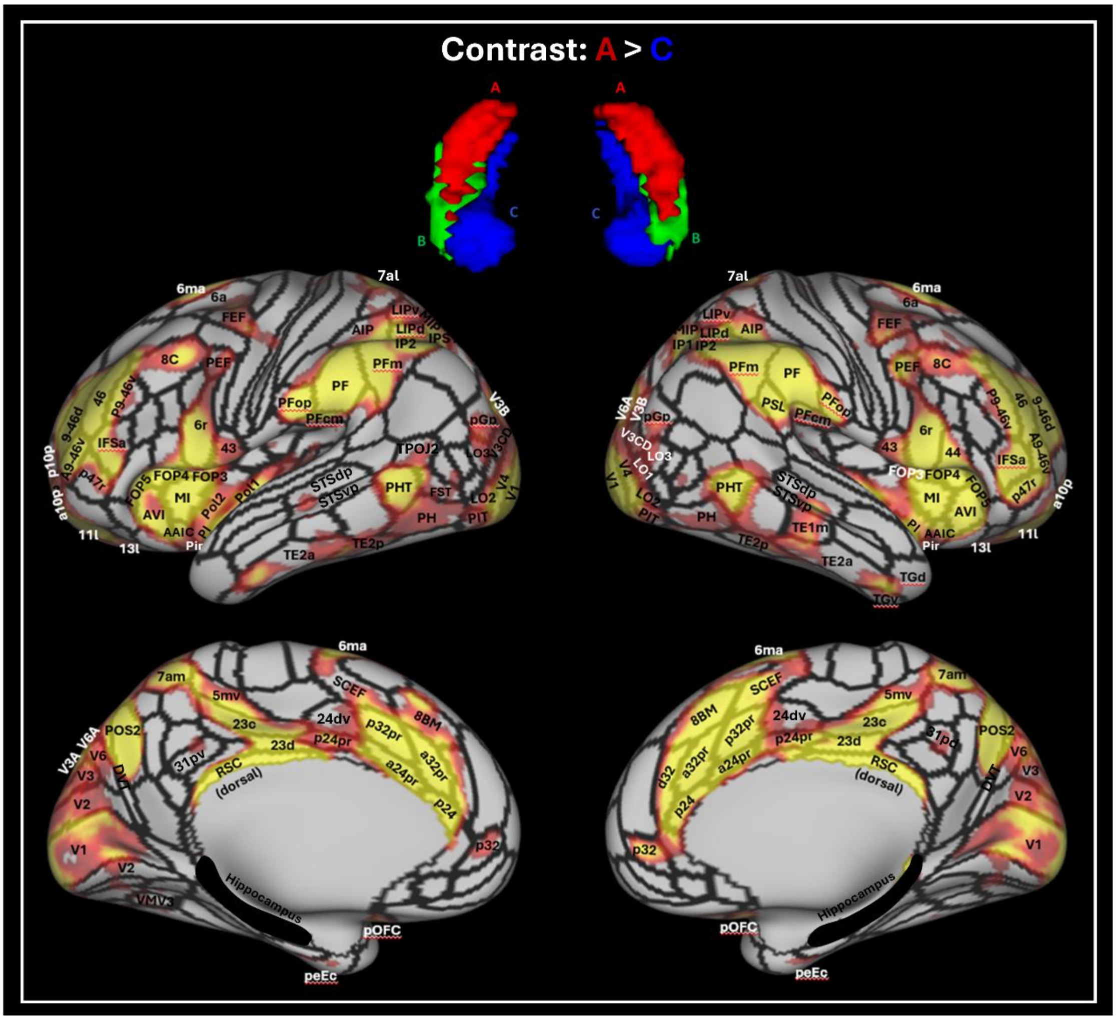

Supplementary Figure 4. ***Results of the seed-based rsfMRI analysis based on the TW-dFC hippocampal clusters.*** *Results for the contrast of cluster A (hippocampal tail cluster) > cluster C (medial cluster). Results are overlaid on the HCPMMP (hippocampus is highlighted in black) and statistically significant areas are labelled. T-test results are thresholded at p<0.05 FDR corrected.*

bioRxiv preprint doi: https://doi.org/10.1101/2025.10.19.683338; this version posted October 20, 2025. The copyright holder for this preprint (which was not certified by peer review) is the author/funder, who has granted bioRxiv a license to display the preprint in perpetuity. It is made available under aCC-BY 4.0 International license.

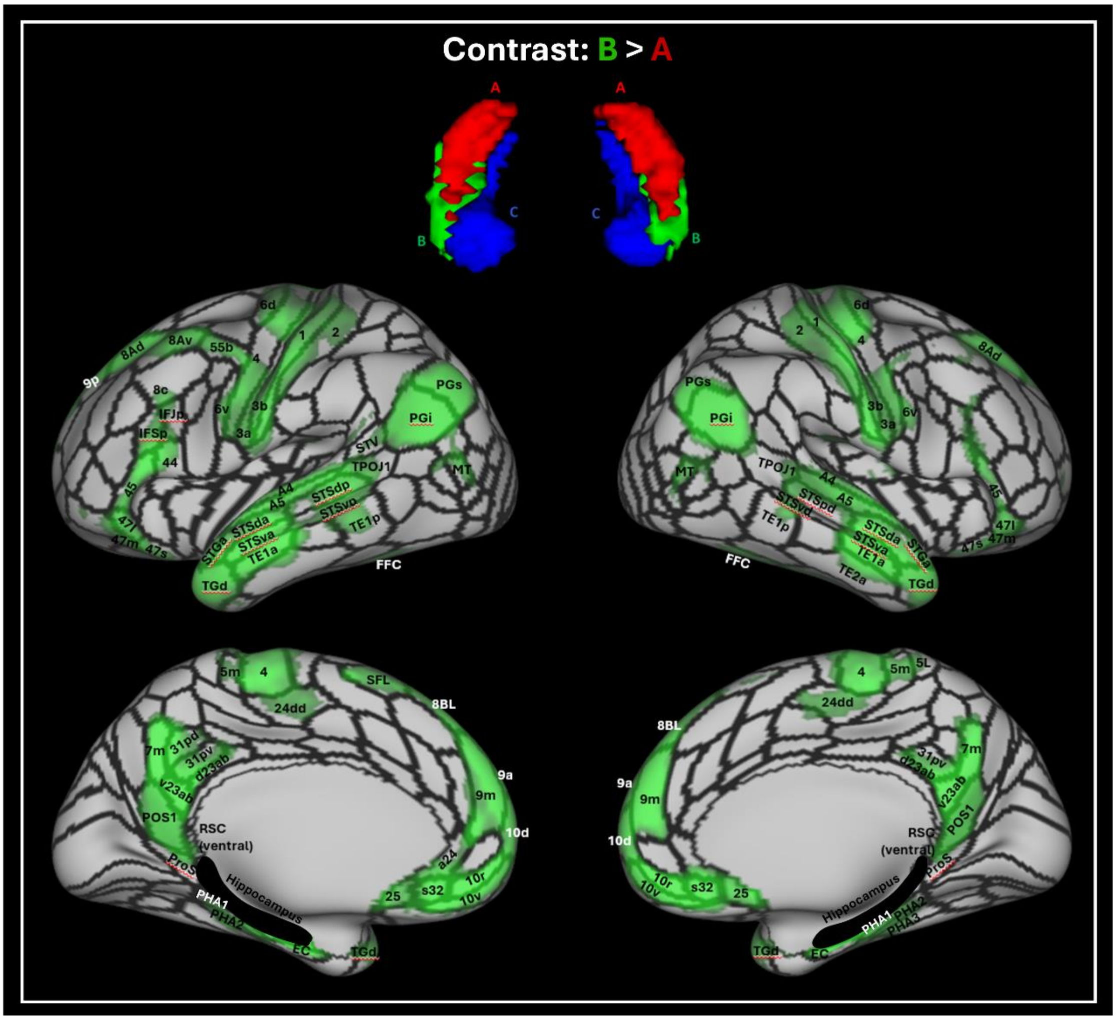

Supplementary Figure 5. *Results of the seed-based rsfMRI analysis based on the TW-dFC hippocampal clusters.* *Results for the contrast of cluster B (anterolateral cluster) > cluster A (hippocampal tail cluster). Results are overlaid on the HCPMMP (hippocampus is highlighted in black) and statistically significant areas are labelled. T-test results are thresholded at p<0.05 FDR corrected.*

bioRxiv preprint doi: https://doi.org/10.1101/2025.10.19.683338; this version posted October 20, 2025. The copyright holder for this preprint (which was not certified by peer review) is the author/funder, who has granted bioRxiv a license to display the preprint in perpetuity. It is made available under aCC-BY 4.0 International license.

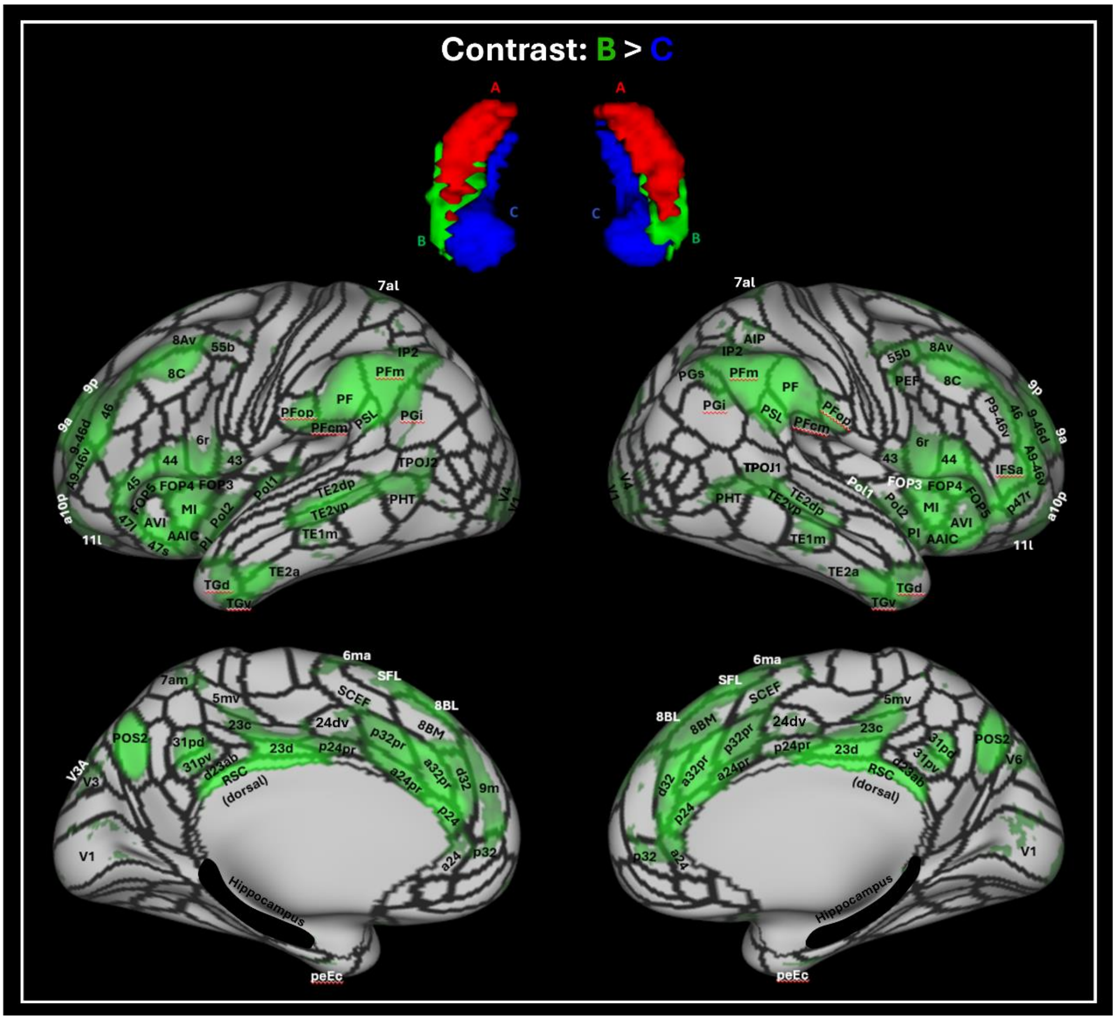

Supplementary Figure 6. *Results of the seed-based rsfMRI analysis based on the TW-dFC hippocampal clusters.* *Results for the contrast of cluster B (anterolateral cluster) > cluster C (medial cluster). Results are overlaid on the HCPMMP (hippocampus is highlighted in black) and statistically significant areas are labelled. T-test results are thresholded at $p<0.05$ FDR corrected.*

bioRxiv preprint doi: https://doi.org/10.1101/2025.10.19.683338; this version posted October 20, 2025. The copyright holder for this preprint (which was not certified by peer review) is the author/funder, who has granted bioRxiv a license to display the preprint in perpetuity. It is made available under aCC-BY 4.0 International license.

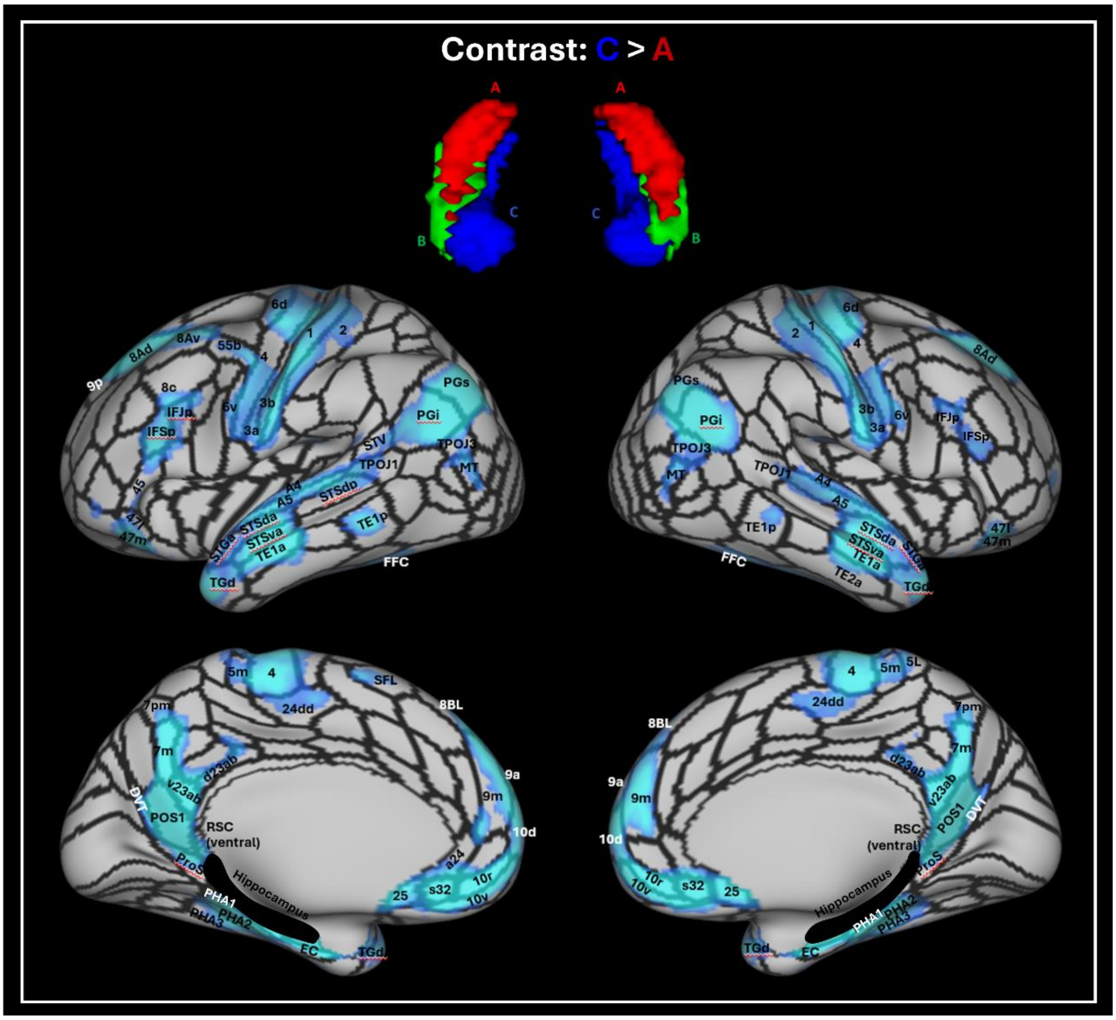

Supplementary Figure 7. ***Results of the seed-based rsfMRI analysis based on the TW-dFC hippocampal clusters.*** *Results for the contrast of cluster C (medial cluster) > cluster A (hippocampal tail cluster). Results are overlaid on the HCPMMP (hippocampus is highlighted in black) and statistically significant areas are labelled. T-test results are thresholded at p<0.05 FDR corrected.*

bioRxiv preprint doi: https://doi.org/10.1101/2025.10.19.683338; this version posted October 20, 2025. The copyright holder for this preprint (which was not certified by peer review) is the author/funder, who has granted bioRxiv a license to display the preprint in perpetuity. It is made available under aCC-BY 4.0 International license.

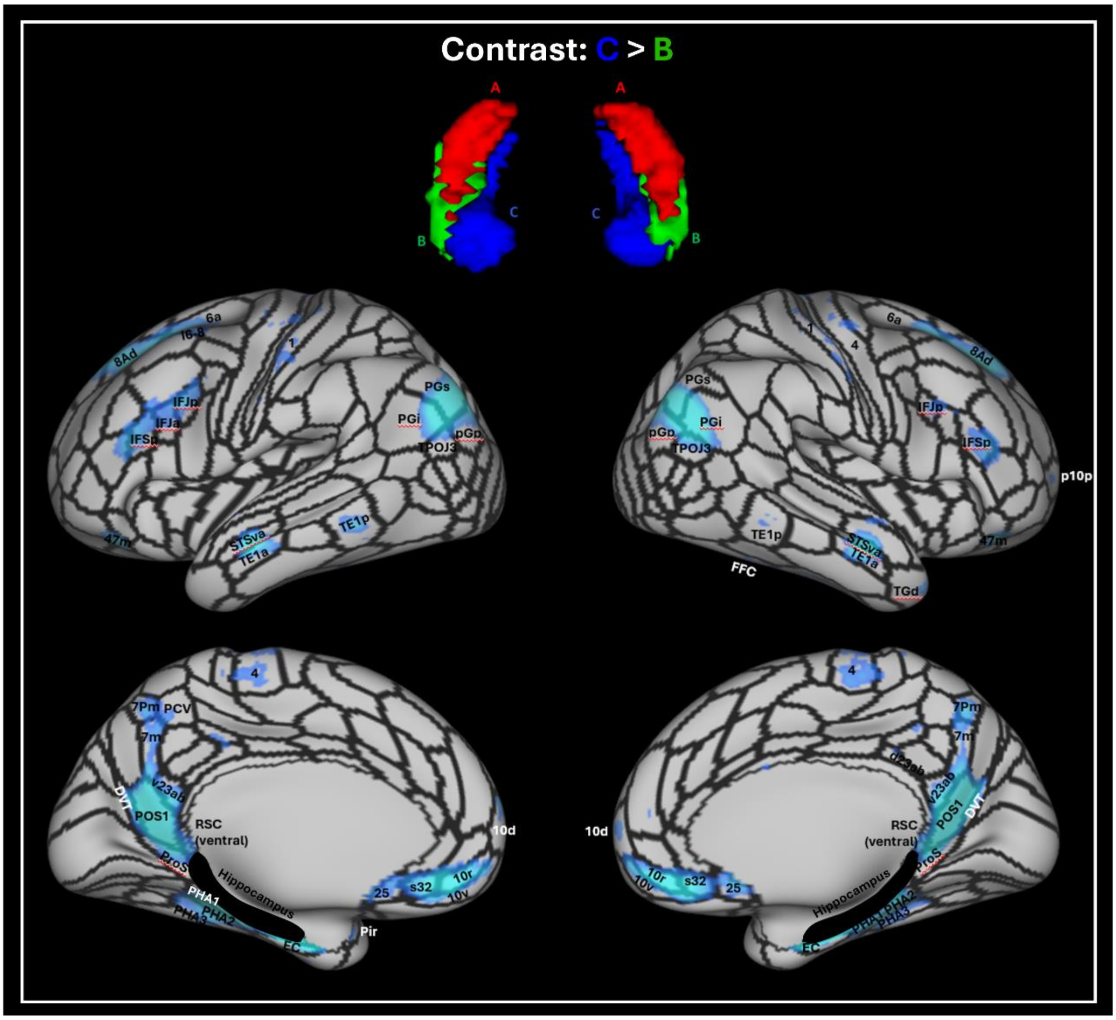

Supplementary Figure 8. ***Results of the seed-based rsfMRI analysis based on the TW-dFC hippocampal clusters.*** *Results for the contrast of cluster C (medial cluster) > cluster B (anterolateral cluster). Results are overlaid on the HCPMMP (hippocampus is highlighted in black) and statistically significant areas are labelled. T-test results are thresholded at p<0.05 FDR corrected.*
